# Adverse Oral Health in Older Adults With Head and Neck Cancer (And Oral Potentially Malignant Disorders): A Systematic Review

**DOI:** 10.1111/ger.70080

**Published:** 2026-05-17

**Authors:** Ciaran Moore, Niamh Walls, Shaima Abujaber, Amanda Willis, Finbarr Allen, Gerry McKenna

**Affiliations:** ^1^ Division of Restorative Dentistry & Periodontology Dublin Dental University Hospital Dublin Ireland; ^2^ Centre for Public Health Queen's University Belfast Belfast UK; ^3^ Centre for Dentistry Queen's University Belfast Belfast UK; ^4^ University College Cork Cork Ireland

**Keywords:** Gerodontology, head and neck cancer, oral potentially malignant disorders, systematic review

## Abstract

**Objective:**

This systematic review evaluated oral health, including oral health‐related quality of life (OHRQoL), in older adults with head and neck cancer (HANC) or oral potentially malignant disorders (OPMDs). The PICO/PECO focus question for this study was: ‘What is the incidence/prevalence of adverse oral health (O) among people aged 60 years or older (P) who have been diagnosed with HANC or an OPMD (E), relative to people aged 60 years or older who have not been diagnosed with HANC or an OPMD, or younger people aged less than 60 years who have been diagnosed with HANC or an OPMD (C)?’

**Methods:**

A comprehensive literature search was performed across PubMed, OVID Medline, CENTRAL and Embase, guided by the PICO/PECO framework. Publications from 1960 up to April 2025 were considered, yielding 1070 records for initial screening.

**Results:**

A total of eight studies were included for analysis. A narrative review only was presented. Findings from the included studies demonstrated conflicting results. Two cross‐sectional studies demonstrated better post‐treatment OHRQoL across some domains for older patients with HANC than younger patients; however, this was contradicted by data from two prospective cohort studies which showed no differences in post‐treatment OHRQoL between older and younger patients with a history of HANC. Findings from a different prospective cohort study demonstrated a linear relationship between HANC patients' age and the incidence of post‐radiotherapy moderate–severe xerostomia. There were no data on oral health consequences among older adults with OPMDs.

**Conclusions:**

This systematic review revealed inconclusive findings on the impact of HANC and OPMD on oral health in older adults.

****PROSPERO Registration:** CRD420251003419:**

## 1 Introduction

Global demographics clearly indicate that the world's population is ageing with an increasing proportion of the overall population comprised of older adults. This demographic transition is driven by a combination of increased life expectancy and declining birth rates. Projections from the United Nations suggest that people aged 60 years and over will double by 2050, reaching approximately 2.1 billion [1]. Global ageing has far‐reaching consequences for various aspects of society, including healthcare systems, social care, labour markets and family structures [2]. While this shift in the distribution of a country's population towards older ages started in high‐income countries, by 2050, two‐thirds of the world's population aged over 60 years will live in low‐ and middle‐income countries [3].

Common conditions in older age include hearing loss, cataracts and refractive errors, back and neck pain and osteoarthritis, chronic obstructive pulmonary disease, diabetes, depression and dementia [4, 5]. As people age, they are more likely to experience several conditions at the same time [6]. Cancer incidence generally increases with age. Most cancers are diagnosed in individuals over 70, and the risk of developing cancer rises significantly after 25–29 years, particularly among females [7]. While early‐onset cancers are increasingly being recognised, they still represent a smaller proportion of overall cases than those diagnosed in older adults. Age‐related changes within the body, including DNA‐damage accumulation and immune system decline, contribute to a higher risk of cancer with age [8].

Head and neck cancer (HANC) is defined by tumour location as a group of malignancies that principally affect the oral cavity, nasal cavity, sinuses, salivary glands, pharynx and larynx [9]. Over 625,000 new cases of HANC are diagnosed worldwide every year [10]. HANC is primarily caused by tobacco and alcohol use, with other risk factors including Human Papilloma Virus (HPV) infection, weakened immune systems and exposure to certain chemicals or radiation [11]. Currently, the most common treatment modalities for HANC are surgery or radiotherapy; however, for more advanced tumours, a combination of surgery and post‐operative radiotherapy, or chemotherapy and radiotherapy, may be required [12]. Approximately, 34%–75% of HANC patients are treated by radiotherapy depending on the location of the tumour [13]. Older people (those over 60 years of age) may have lower survival and greater morbidity after treatment for HANC than younger patients, but the evidence is conflicting [14, 15, 16, 17].

Oral potentially malignant disorders (OPMDs) are a diverse group of conditions defined as ‘any oral mucosal abnormality that is associated with a statistically increased risk of developing oral cancer’ [18]. Globally, OPMDs account for 4.5% of oral lesions and are associated with the use of tobacco, alcohol and the areca nut [19]. They can be classified into high‐risk lesions such as erythroplakia, non‐homogeneous leukoplakia, proliferative verrucous leukoplakia and actinic keratosis, and those of lower or undefined risks such as homogeneous leukoplakia, oral submucous fibrosis and oral lichen planus. The risk of malignancy in a patient with an OPMD is variable and is dependent on several factors including clinical presentation, histological subtype, location, size and patient demographics, and has been estimated to be 7.9% for all types of OPMDs [20]. Malignant transformation is known to occur slowly—over many years—but does not occur in all cases. Typically, OPMDs present as white and/or red patches with variable margins and surfaces ranging from exophytic, wrinkled or corrugated. Depending on the nature of the OPMD, other features may be apparent such as ulceration, loss of pigmentation, loss of tongue papillae, leathery mucosa, fibrous bands, limited tongue mobility and mouth opening [21]. The gold standard method for diagnosing OPMDs is through biopsy, whilst management entails assessment of risk, educating patients on features which could indicate malignant transformation, counselling on risk factors and long‐term regular review. Chemo‐prevention trials have been complicated by methodological limitations and adverse events [22]. High‐risk lesions, where possible, should be excised in the absence of any surgical complications [23].

The oral side‐effects of HANC treatments such as radiotherapy and chemotherapy have been well documented. HANC patients are at higher risk of post‐radiotherapy dental caries and periodontitis due to radiotherapy‐mediated salivary gland hypofunction, as well as radiation damage to dental and periodontal hard tissues [24, 25, 26]. Approximately one‐third of patients develop post‐radiotherapy dental caries [27], and post‐radiotherapy periodontitis has been shown to affect 18%–68% of HANC patients [28, 29, 30, 31, 32, 33]. Moreover, patients treated with head and neck radiotherapy or chemotherapy are more likely to develop hyposalivation (decreased salivary flow) and xerostomia (the subjective feeling of dry mouth), oral mucositis, and impaired oral health‐related quality of life (OHRQoL) [34, 35, 36, 37, 38]. In addition, surgical excision of a locally advanced tumour may also result in severe anatomical disfigurement, loss of teeth and extensive scarring and permanent changes to the structure and function of various tissues within the head and neck [24, 39]. Previous research into OPMDs has also suggested possible associations between disease and xerostomia, periodontitis and impaired OHRQoL, particularly among patients affected by oral lichen planus or oral submucous fibrosis [40, 41, 42, 43, 44].

The aim of this systematic review was to determine the impact of HANC or OPMD and their management on oral health in older adults compared to both older adults without a diagnosis of HANC or OPMD and younger patients with HANC or OPMD.

## 2 Materials and Methods

This systematic review was carried out and reported on in accordance with the PRISMA (Preferred Reporting Items for Systematic Reviews and Meta‐Analyses) guidelines [45, 46]. The protocol for this systematic review was registered via PROSPERO (the International Prospective Register of Systematic Reviews) under the registration number CRD420251003419.

### 2.1 Eligibility Criteria and Information Sources

Details of the inclusion and exclusion criteria for this review along with the relevant sources of information used to explore pertinent records are described in Table 1. The final literature search was performed on 4th April 2025.

**TABLE 1:** Search strategy and selection criteria.

Focus question	What is the incidence/prevalence of adverse oral health (O) among patients aged 60 years or older (P) that have been diagnosed with head and neck cancer or oral potentially malignant disorder (OPMD) (E) compared to patients aged 60 years or older that have not been diagnosed with HANC or OPMD, or younger patients aged less than 60 years that have been diagnosed with HANC or an OPMD (C)?
Population	Studies involving older adults (patients aged 60 years and over)
Intervention/Exposure	Patients diagnosed with malignant neoplasm of the lip, oral cavity or pharynx (as defined by codes 2B6 in the International Classification of Diseases version 11) or oral potentially malignant disorders (e.g., erythroplakia and leukoplakia)
Comparison	Studies that have made comparisons with groups of patients that have not been diagnosed with head and neck cancer or potentially malignant oral lesions or patients that are younger than 60 years and that have been diagnosed with head and neck cancer or oral potentially malignant disorder. Please note that a comparator is not an essential criterion for a study's inclusion in this systematic review.
Outcome	The outcomes measures of interest for this systematic review include: • Dental caries • Gingivitis and periodontitis • Tooth mobility and tooth loss • Peri‐apical infection • Hyposalivation and xerostomia • Oral health related quality of life including objective (e.g., OHRQoL, global health rating) and subjective outcomes (e.g., pain, function and speech performance)
Language	No restriction
Search date	4th April 2025. Publications from 1960 up to April 2025 were considered.
PROSPERO	Registration 12th March 2025
*Database search, no language restriction*	
Databases	MEDLINE (Ovid), Embase, PubMed, The Cochrane Library
*Supplementary handsearch*	
Journals	The reference lists of all included studies will be checked for additional relevant research articles.
References	Included articles and identified reviews
*Grey literature search*	
Conference proceedings	Not applicable
Contact with experts	Not applicable
Study registers	Not applicable
*Selection process*	
Inclusion criteria	Clinical investigations of any study design related to the focus question Confirmed diagnosis of head and neck cancer or OPMD
Contact with authors	Research potentially met the inclusion criteria, but full text article was unavailable Research potentially met the inclusion criteria, but data reporting was incomplete or unclear
Exclusion criteria	Topic not relevant to the focus question Aggregated oral health data not stratified by patient age Reviews In vitro study Animal study Insufficient participant information and no response from investigators when seeking clarification Previous investigations reporting on the same patient population (excluded but retained for reference)
Identification process	Records were reviewed by at three investigators independently, disagreements were resolved by discussion, and authors were contacted for clarification when required

### 2.2 Search Strategy and Selection Process

The search terms were developed using the PI/ECO (Population, Intervention/Exposure, Comparison and Outcome) framework and combined with Boolean operators (OR, AND, and NOT) to construct the search strategy. Please note that the presence of a comparator group was not deemed an essential criterion for a study's inclusion in this systematic review. The first reviewer (C.M.) performed the initial search across all specified databases. Any issues identified during the process were corrected and the search strategy was refined accordingly. Details of all search terms and the final search strategy are provided in Table 2.

**TABLE 2:** Final search strategy. [Colour table can be viewed at wileyonlinelibrary.com]

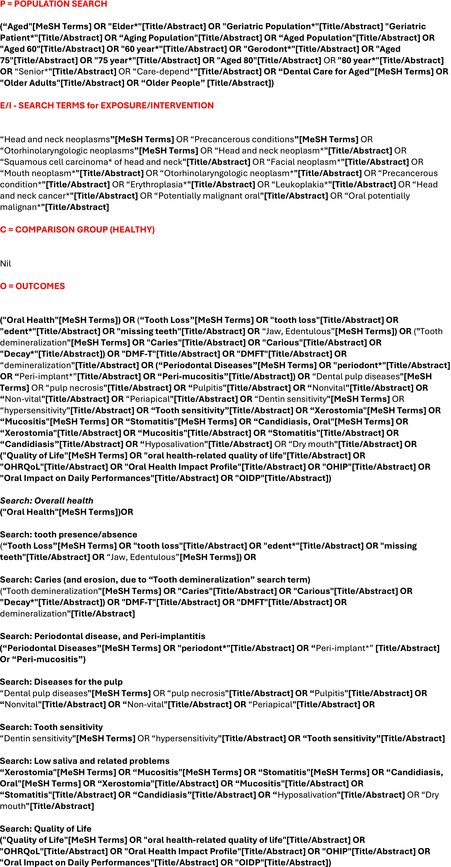

Search results from each database were imported into EndNote. The software automatically removed duplicate records as an initial step. Following this, C.M. and two additional investigators (N.W. and S.A.) independently screened titles and abstracts within the platform. Following this, selected studies underwent full‐text review and data extraction based on mutual agreement between the three investigators.

When full text articles were not available, or lacked sufficient detail, the reviewers contacted the corresponding author in an attempt to secure additional details. Reference cross‐checks and manual searches were also performed to identify studies that were not discovered via the search strategy.

### 2.3 Data Collection Process and Data Items

Relevant information collected from eligible articles included: study design, number of participants, participant characteristics, age profile of participants, oral health outcome assessment methods and oral health outcome measures such as xerostomia and OHRQoL. The main findings from each study were summarised focusing on the review's initial research question.

### 2.4 Study Risk of Bias

The risk of bias was assessed using the Cochrane Collaboration's ‘Risk of bias assessment tool – version 2 (RoB 2)’ for randomised trials [47]. Non‐randomised studies were assessed using the ‘Risk of bias in non‐randomised studies – of exposure (ROBINS‐E)’ [48], with the methodological quality of non‐randomised studies also assessed using the Newcastle‐Ottawa Scale (NOS) for cohort studies and cross‐sectional studies [49, 50].

### 2.5 Summary Measures and Synthesis of Results

It was anticipated in advance of this systematic review that there would be insufficient quality data from included studies to facilitate formal data synthesis through meta‐analysis, and so a narrative review only was conducted.

## 3 Results

### 3.1 Study Selection

The search strategy detected a total of 1480 records [MEDLINE (PubMed): *n* = 577; MEDLINE (OVID): *n* = 231; Embase: *n* = 658; CENTRAL: *n* = 14]. The removal of duplicated records resulted in the elimination of 410 records. A total of 1070 titles and abstracts were screened. Forty‐seven full text relevant records were identified and retrieved, along with two additional articles identified by a manual reference search of these 47 records. Therefore, a final list of 49 articles was full text reviewed, of which 41 were excluded based on the following criteria—no objective or subjective measures of oral health: *n* = 7; aggregated oral health data presented across all age groups: *n* = 17; follow‐up less than 6 months: *n* = 3; PI/ECO not clearly defined: *n* = 10; orodigestive cancer/non‐HANC: *n* = 2; review article: *n* = 1; study presented duplicated data to an already included article: *n* = 1.

Finally, a total of eight studies with relevant data on oral health in older adults with HANC were included [51, 52, 53, 54, 55, 56, 57, 58]. There were no research studies identified that had specifically investigated the incidence or prevalence of oral health among older adults with OPMDs. Of the 8 included studies, four were prospective or retrospective observational cohort studies [51, 52, 53, 58], three were cross‐sectional in nature [55, 56, 57] and one study was a randomised controlled trial [54] (Table 3).

**TABLE 3:** Summary of information from included studies.

Author	Type of study	Year of publication	Number of patients	Age (years)	Follow‐up (months)	Outcome	Outcome assessment	Site of head and neck cancer	Head and neck cancer treatment	Oral health impact (OHRQoL)
Aoki et al.	Prospective cohort study	2021	172	43 patients in the older adult group (greater than or equal to 75 years)—median age = 80 years, and 129 patients under 75 years of age (range 21–74 years)—median age = 62 years	1‐, 3‐ and 6‐months	Quality of life	Functional Assessment of Cancer Therapy – Head and Neck version 4 scale (FACTH& N)	Oral cavity cancer	No statistically significant difference in treatment modalities used for older vs. younger group. All patients had surgery; 2% had adjuvant radiotherapy.	No statistically significant differences in OHRQol between the older and younger age groups. Both age groups of patients reported an initial deterioration in the domain of swallowing; however the majority of these issues were resolved 6 months after treatment.
Beetz et al.	Prospective cohort study	2013	307	103 patients aged greater than 65 years, and 204 patients aged 65 years or younger. Overall mean age = 62 years	6‐, 12‐, 18‐ and 24‐months post‐treatment	Quality of life (xerostomia)	EORTC QLQ‐H&N35	Primary HANC any region of head and neck	Curative radiotherapy either alone (67%), or in combination with chemotherapy or cetuximab (no age‐related breakdown).	A linear relationship between greater patient age and risk of ‘moderate–severe’ xerostomia after radiotherapy. Older patients also had an increased risk of moderate–severe xerostomia at 6‐, 12‐, 18‐ and 24‐months post‐treatment. Older patients had a significantly reduced rate of recovery regarding the sensation of dry mouth 6‐, 12‐, 18‐ and 24‐months post‐radiotherapy than patients less than 65 years.
Derks et al.	Prospective cohort study	2004	121	51 ‘older’ (greater than or equal to 70 years), and 70 ‘younger’ patients (45–60 years)	3‐, 6‐ and 12‐months	Quality of life	EORTC QLQ‐C30, QLQ‐H&N35	Squamous cell carcinoma of the oral cavity, oropharynx and hypopharynx, or larynx without distant metastasis	Statistically significant differences in treatment modality used—31% of older patients had surgery + radiotherapy vs. 60% of younger patients; 38% of older patients had surgery only vs. 11% of younger patients; 23% of older patients had radiotherapy only vs. 11% of younger patients; and 4% of older patients had chemo‐radiotherapy vs. 16% of younger patients.	No statistically significant differences in the OHRQoL domains of: (i) teeth, (ii) mouth opening, (iii) dry mouth and (iv) sticky saliva, between the older and younger age groups of patients at 3‐, 6‐ and 12‐months follow‐up.
Lafont et al.	Randomised controlled trial	2024	475 (GA‐driven interventions and follow‐up (intervention group, *n* = 238) or standard of care (control group, *n* = 237))	All patients greater than or equal to 65 years	6‐, 12‐ and 24‐months post‐intervention	Quality of life	EORTC QLQ‐C30, QLQ‐H&N35	Head and neck cancer—any region of the head and neck	51%–55% of patients underwent surgery; 14%–15% underwent chemo‐radiotherapy; 13%–14% underwent radiotherapy only.	No statistically significant differences in mean scores regarding OHRQoL domains between the intervention and control groups. Overall, the GA‐assessment driven interventions were found to have no effect on post‐treatment OHRQoL for older head and neck cancer patients.
Laraway et al.	Cross‐sectional study	2012	638	Four subgroups defined by age (up to 55 years—177, 55–64 years—217, 65–74 years—165, 75 years and over—79)	12 months after primary surgery or cancer diagnosis (range 12–30 months)	Quality of life	University of Washington Head and Neck Quality of Life scale	Primary SCC any region of head and neck	Majority had surgery only (63%–72%) with no significant difference in type of treatment across the age groups. 28%–35% had surgery + radiotherapy‐ or chemotherapy; 2% had radiotherapy or chemotherapy only.	People older than 65 years reported better functional outcomes of speech, swallowing, and saliva, 12‐months after their cancer diagnosis or primary surgery than younger patients. In relation to patient‐reported salivary flow, the best scores were seen in patients aged 75 years and over. In relation to speech, there was a progression towards better scores in those aged 65–74 years, and scores were better still for those aged 75 years and over.
Linsen et al.	Cross‐sectional study	2018	1319	674 (51.1%) patients were 45–60 years, and 645 (48.9%) were 61–100 years	At least 6 months	Quality of life	The Bochum Questionnaire on Rehabilitation	Oral cavity squamous cell carcinoma	All patients underwent oncologic surgery. Besides surgical therapy, 53.9% of the younger and 44.9% of the older patients (61–100 years old) received additional radiotherapy, while 20.2% of the younger and 18.7% of the older patients received additional chemotherapy. Neck dissection was performed in 51% of the younger and 37.2% of the older patients.	Patients aged over 60 years were found to have lost significantly less teeth and reported less oral and temporomandibular joint pain greater than 6 months after cancer treatment than patients aged 45–60 years. Patients aged 60 years or older also had a lower prevalence of severe eating and swallowing issues than patients aged 45–60 years; however, there were no statistically significant differences in the reported prevalence of dry mouth, taste disturbance, and limited mouth opening between patients aged 60 years or older and those aged 45–60 years.
Ruhle et al.	Cross‐sectional study	2021	50	All patients greater than or equal to 65 years. Median age = 69 years	Median time between radiotherapy and outcome assessment was 32 months (range 12–113 months)	Quality of life	EORTC QLQ‐C30, QLQ‐H&N35	Head and neck squamous cell carcinoma of any region of head and neck	Twenty‐three patients (46%) underwent definitive (chemo‐) radiotherapy, while 27 patients (54%) received (chemo‐) radiotherapy after primary surgery.	Older patients (aged 65 years or older) 1 to 10 years post‐radiotherapy were determined to have comparable global quality of life to equivalently aged people from the general German population, except in the domain of social functioning. They also found no differences in self‐reported oral health (as determined by EORTC QLQ‐H&N35 oral health related domains) between the Older head and neck cancer patient population and the equivalent aged general German population.
Ruhle et al.	Retrospective cohort study	2021	29	All patients greater than or equal to 65 years. Median age = 75 years	Range 3–60 months	Chronic treatment related toxicity	Common Terminology Criteria for Adverse Effects (CTCAE) version 5.0	Primary salivary gland carcinoma	More than two‐thirds (*n* = 20, 69.0%) of patients were treated with adjuvant (chemo‐) radiotherapy after surgery +9 patients were treated with definitive (chemo)radiotherapy (31.0%).	The incidence of post‐treatment chronic toxicity as assessed by CTCAE 3–60 months post‐treatment was deemed to be low.

Abbreviation: OHRQoL, oral health related quality of life.

**FIGURE 1:**
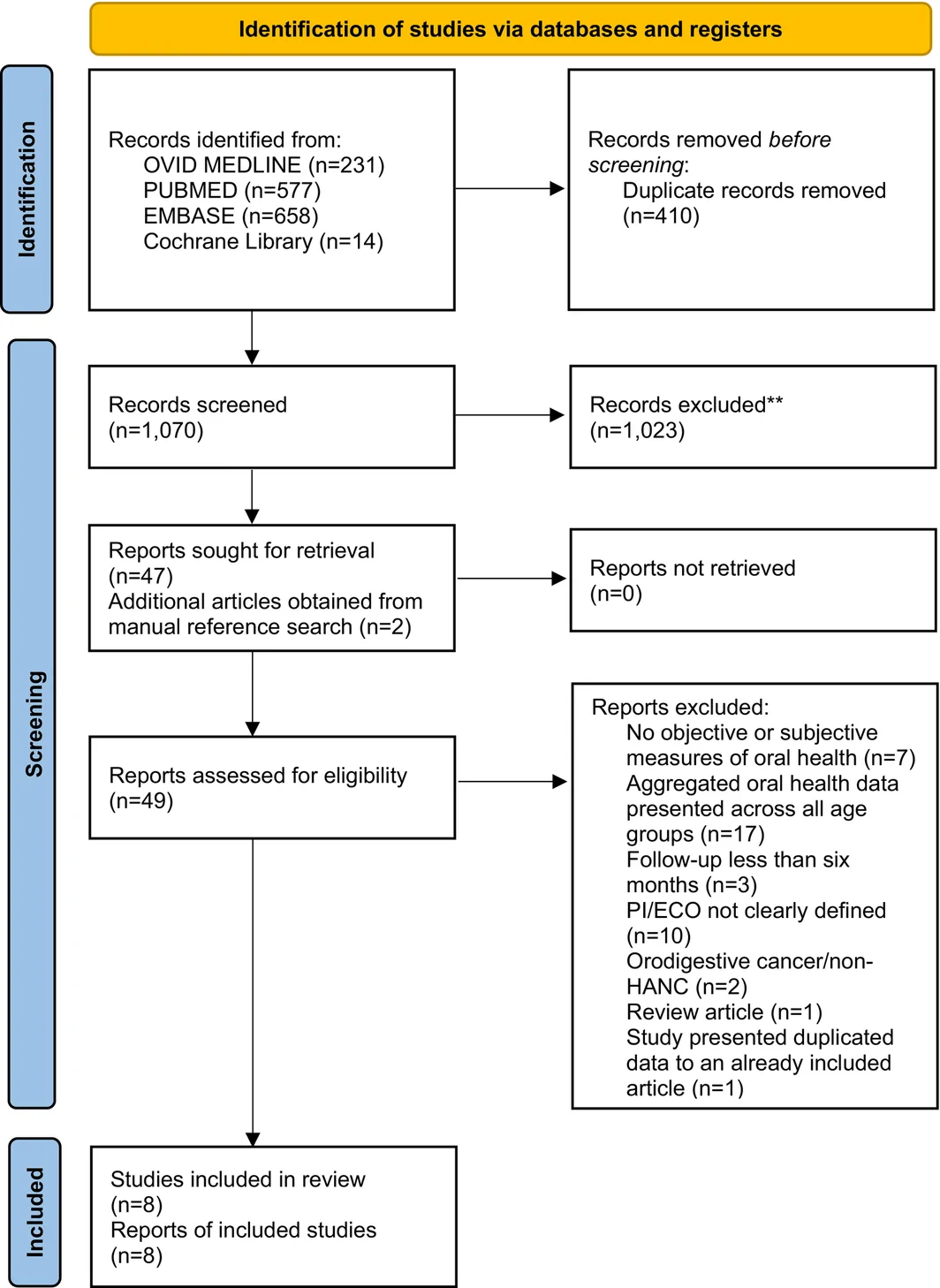
PRISMA flow diagram. [Colour figure can be viewed at wileyonlinelibrary.com]

The study selection process is detailed via a PRISMA flow diagram—Figure 1. The reasons for the exclusion of 41 articles are described in Appendix S1.

### 3.2 Study Characteristics

Five of the eight included studies recruited people with cancer affecting any region of the head and neck [52, 53, 54, 55, 57]. Two studies analysed participants with oral cancer only [51, 56], and one study assessed patients with primary salivary gland cancers only [58]. Five studies explored differences in oral health based on patient age [51, 52, 53, 55, 56], and three studies recruited people aged 65 years or over only [54, 57, 58].

All of the eight included studies recruited participants that had undergone surgery, radiotherapy, or chemotherapy, or a combination of these treatments [51, 52, 53, 54, 55, 56, 57, 58]. Seven of the eight studies assessed OHRQoL using dedicated validated patient‐administered questionnaires (EORTC QLQ‐C30: *n* = 4 [52, 53, 54, 57]; Functional Assessment of Cancer Therapy—Head and Neck version 4 scale: *n* = 1 [51]; University of Washington Head and Neck Quality of Life scale: *n* = 1 [55]; The Bochum Questionnaire on Rehabilitation: *n* = 1 [56]) and one study assessed chronic treatment‐related toxicity using the Common Terminology Criteria for Adverse Effects (CTCAE) version 5.0 [58].

### 3.3 Synthesis of Results: Descriptive Analysis of the Included Studies

Owing to substantial disparity in the methodologies of the included studies, it was deemed inappropriate to combine results via meta‐analysis, and so a narrative review only is presented.

In a cross‐sectional study of 638 HANC patients, Laraway et al. [55] found that people older than 65 years reported better functional outcomes of speech, swallowing and saliva, 12 months after their cancer diagnosis or primary surgery than younger patients. In relation to patient‐reported salivary flow, the best scores were seen in patients aged 75 years and over. In relation to speech, there was a progression towards better scores in those aged 65–74 years, and scores were better still for those aged 75 years and over. Approximately 7%–8% of patients over 65 years reported serious problems with swallowing, while 3%–6% of patients over 65 years reported serious issues with speech (both significantly less than patients under 65 years). 9%–11% of patients over 65 years reported serious issues with chewing, 6%–9% reported serious issues with taste, and 6%–14% reported serious issues with salivary flow; however, there were no statistically significant differences in these outcomes over those aged under 65 years [55].

Similarly, in a cross‐sectional study of 1319 oral cancer patients by Linsen et al. [56], patients aged over 60 years were found to have lost significantly fewer teeth and reported less oral and temporomandibular joint pain greater than 6 months after cancer treatment than patients aged 45–60 years. 52.7% of older patients (61–100 years old) had lost at least one tooth during oncology treatment, while it was 75.7% among younger patients. Moreover, 16.4% of older patients (but 21.9% of younger patients) reported oral pain; for temporomandibular joint pain it was 17.1% and 23.2%, respectively. Patients aged 60 years or older also had a lower prevalence of severe eating and swallowing issues (27.2%) than patients aged 45–60 years (34.4%); however, there were no statistically significant differences in the reported prevalence of dry mouth (26.4% vs. 30.9%), taste disturbance (13.4% vs. 15.9%), and limited mouth opening (19.3% vs. 22.3%) between patients aged 60 years or older and those aged 45–60 years [56].

In a cross‐sectional study of 50 people by Ruhle et al. [57], older patients (aged 65 years or older) 1–10 years post‐radiotherapy were determined to have comparable global quality of life to equivalently aged people from the general German population, except in the domain of social functioning. They also found no differences in self‐reported oral health (as determined by EORTC QLQ‐H&N35 oral health related domains) between the older HANC patient population and the equivalent aged general German population. The majority of EORTC QLQ‐H&N35 symptom subscales showed comparable score values between the definitive and adjuvant radiotherapy subgroups; however, patients who did not require primary tumour resection reported fewer symptoms such as ‘troubles with social eating’ (*p* < 0.05) and mouth opening (*p* < 0.01) than patients who underwent primary surgery. Moreover, patients treated with radiotherapy only had significantly fewer long‐term issues with swallowing than patients treated with chemo‐radiotherapy [57].

By contrast, in a prospective cohort study of 307 patients with HANC, Beetz et al. [52] observed a linear relationship between greater patient age and the occurrence of ‘moderate–severe’ xerostomia (defined by patients rating ‘quite a bit’ or ‘a lot of dry mouth’ via the EORTC QLQ‐H&N35 questionnaire) after radiotherapy. Older patients also had a higher risk of moderate–severe xerostomia at 6, 12, 18 and 24 months post‐treatment, irrespective of baseline xerostomia or whether QUANTEC criteria for radiotherapy dose limitation to the parotid glands were met (at least one parotid gland irradiated to a mean dose of less than 20 Grey, or both glands collectively irradiated to a mean dose of less than 25 Grey). Furthermore, older patients had a significantly lower rate of recovery with the sensation of dry mouth 6, 12, 18 and 24 months post‐radiotherapy than patients younger than 65 years. Approximately 10%–45% of patients aged 65 years or older reported moderate–severe xerostomia 6–24 months post‐treatment where QUANTEC criteria were met and where patients reported no baseline xerostomia; this was 25%–75% where QUANTEC criteria were met and where patients reported ‘minor’ baseline xerostomia; 45%–85% where QUANTEC criteria were not met and where patients reported no baseline xerostomia; and 70%–95% where QUANTEC criteria were not met and where patients reported ‘minor’ baseline xerostomia [52].

Derks et al. [53] compared OHRQoL among post‐treatment HANC patients aged 70 years or over (*n* = 51) and those aged 45–60 years (*n* = 70). They found no statistically significant differences in the OHRQoL domains of: (i) teeth, (ii) mouth opening, (iii) dry mouth and (iv) sticky saliva between the older and younger age groups of patients at 3‐, 6‐ and 12‐month follow‐up. Both older and younger patients reported a deterioration in OHRQoL scores specifically for mouth opening, dry mouth and sticky saliva from baseline to post‐treatment. Furthermore, patients reported minimal recovery in the sensations of dry mouth and sticky saliva between 3‐, 6‐ and 12‐months post‐treatment; however, there was more substantial recovery in mouth opening between 3‐, 6‐ and 12‐months post‐treatment [53].

Similarly, Aoki et al. [51] compared OHRQoL among post‐surgical oral cancer patients aged 75 years or over (*n* = 43) and those aged 21–74 years (*n* = 129). They found no statistically significant differences in OHRQoL between the older and younger age groups. Both age groups reported an initial deterioration in the domain of swallowing; however, the majority of these issues had been resolved by 6 months after treatment [51].

In a retrospective cohort study of 29 patients with primary salivary gland cancer aged 65 years or older by Ruhle et al. [58], the incidence of post‐treatment chronic toxicity as assessed by CTCAE 3–60 months post‐treatment was deemed to be low. Three patients (15.8%) suffered from grade 3 (severe) toxicity—one patient with chronic pain due to osteoradionecrosis, one patient with mucositis and oral thrush and one patient with chronic lymphoedema. Three (15.8%) patients were determined to have grade 2 (moderate) dry mouth [58].

Lafont et al. [54] undertook a randomised controlled trial of 475 HANC patients aged 65 years or older that aimed to assess the effects of geriatric assessment (GA)‐driven interventions on patients' quality of life over 2 years post‐treatment. Patients in the interventional group received a four‐component, GA‐driven intervention and were followed up by a geriatrician during cancer treatment and up to 24 months after randomisation (comprising of 7 scheduled consultations that entailed: (i) optimising the management of health problems detected during the GA; (ii) educating the patient about the self‐management of comorbidities; (iii) providing information about cancer treatments; and (iv) performing a medication review). Patients from both the interventional and control groups reported a deterioration in speech, social eating, swallowing and dry mouth, 6 months after oncology treatment. Mean scores in the domains of social eating and swallowing returned to baseline levels 12–24 months post‐treatment for both groups, but mean scores in the domains of speech and dry mouth did not recover to baseline levels 24 months post‐oncology treatment for patients from either group. There were no statistically significant differences in mean scores for OHRQoL domains between the intervention and control groups. Overall, the GA‐assessment driven interventions were found to have no effect on post‐treatment OHRQoL for older HANC patients [54].

### 3.4 Risk of Bias and Quality Assessment of the Included Studies

**FIGURE 2:**
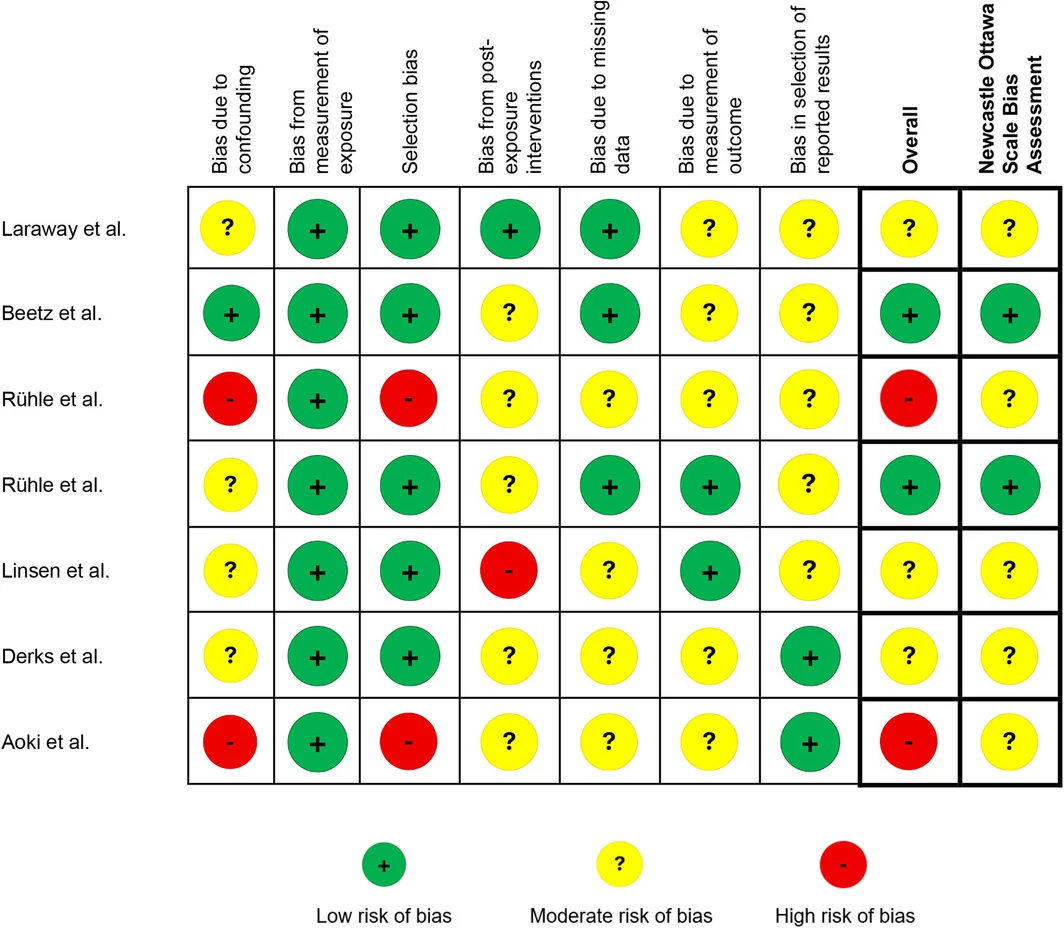
Risk of bias summary of non‐RCTs using Cochrane Collaboration ROBINS‐E tool and the Newcastle Ottawa Scale. [Colour figure can be viewed at wileyonlinelibrary.com]

**FIGURE 3:**
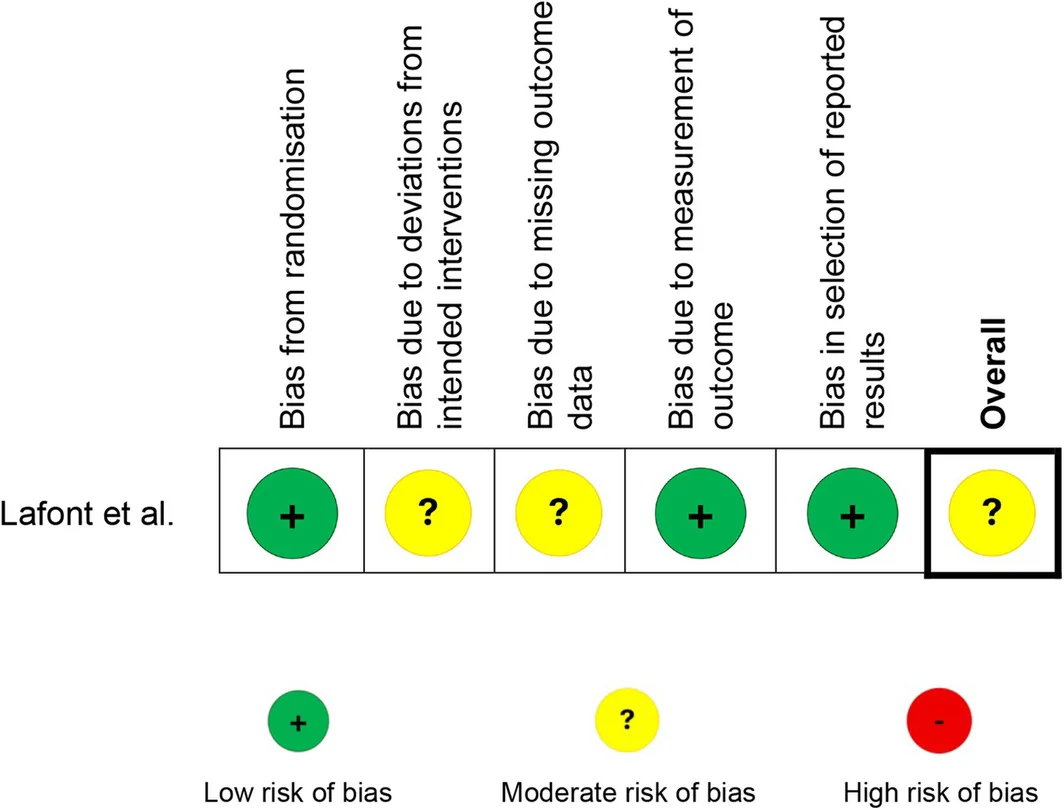
Risk of bias summary of RCTs using Cochrane Collaboration RoB 2 tool. [Colour figure can be viewed at wileyonlinelibrary.com]

The risk of bias assessments for the included studies are presented graphically in Figures 2 and 3. Two observational studies were deemed to have low risk of bias [52, 58], and two studies were assessed as having a high risk of bias using the Cochrane Collaborations ROBINS‐E tool and a moderate risk of bias using the Newcastle Ottawa Scale assessment tool [51, 57]. The three remaining observational studies were deemed to have a moderate risk of bias/‘some concerns’ [53, 55, 56]. The randomised controlled trial conducted by Lafont et al. [54] was determined to have a moderate risk of bias/‘some concerns’ as assessed by the Cochrane Collaboration's RoB 2 tool.

## 4 Discussion

This systematic review aimed to explore the impact of HANC and OPMDs on oral health in older adults. A total of eight studies relevant to HANC were eligible for inclusion; however, no studies on OPMDs were identified. This demonstrates the lack of previous research within this field, and in particular, the dearth of oral health and oral health related quality of life (OHRQoL) data for older adult patients with HANC or OPMDs.

The findings from the eight included studies demonstrated conflicting findings. The cross‐sectional studies conducted by Laraway et al. and Linsen et al. revealed that older patients reported better oral health and OHRQoL across some domains 6–12 months after their oncology treatment than younger patients [55, 56]. Data from prospective cohort studies conducted by Aoki et al. and Derks et al., however, found no differences in oral health and OHRQoL between older and younger patients up to 12 months after HANC treatment [51, 53]. Beetz et al. demonstrated a linear relationship between patient age and the incidence of post‐radiotherapy moderate–severe xerostomia at 6, 12, 18 and 24 months after treatment, and irrespective of parotid‐gland sparing or baseline sensation of dry mouth [52].

Given the limited number of studies included, the small sample sizes in each study, the fact that seven out of the eight studies were observational in nature (including three cross‐sectional studies), and that six studies were deemed to be at moderate‐high risk of bias, it is difficult to draw any meaningful conclusions from this review in relation to oral health in older patients with HANC. In addition, none of the eight included articles presented data relating to objective, clinician‐led measures of oral health, with all data derived by subjective assessment or patient‐administered questionnaires. A total of 17 out of 41 articles (including 4 that had assessed oral health in patients with OPMDs) identified by the search strategy and that contained potentially relevant data were excluded as they had presented summary oral health statistics that collated data across all groups without further age‐specific subgroup analyses.

The findings from all eight studies demonstrated a deterioration in patients' oral health after HANC treatment. Patients treated for HANC have previously been shown to have a higher risk of dental caries, periodontitis, xerostomia and hyposalivation, mucositis and limited mouth opening, along with poorer quality of life [24, 25, 26, 27, 34, 55, 59, 60, 61]. Approximately one‐third of patients develop dental caries within 2 years of head and neck radiotherapy due to the direct and indirect effects of radiation treatment [27]. A previous systematic review and meta‐analysis by Jensen et al. also estimated that xerostomia affects 74% of HANC patients 1–3 months post‐radiotherapy, and 85% of HANC patients greater than 2 years post‐radiotherapy [34]. Moreover, oral and oropharyngeal mucositis can affect up to 80% of patients, and a previous systematic review and meta‐analysis determined a combined weighted prevalence of trismus of 25% for patients treated with conventional radiotherapy, while it was 5% for patients treated with intensity‐modulated radiotherapy (IMRT) [61, 62].

Previous research in relation to oral health in patients treated for OPMDs is much more limited. Patients with OPMDs may have a higher risk of periodontal disease, hyposalivation and xerostomia, and impaired OHRQoL [40, 41, 42, 43, 44]; however, more research is needed in this field. Future research should prioritise longitudinal studies aimed at analysing both objective and subjective measures of oral health in older adults with HANC as well as older adults with OPMDs. These studies should use standardised and validated assessment tools for OHRQoL such as the Oral Health Impact Profile (OHIP) or EORTC QLQ [63, 64], as well as objective measures of oral health that utilise recognised established methodologies such as the International Caries Detection and Assessment System (ICDAS) for dental caries [65].

## 5 Conclusions

This systematic review revealed inconclusive findings on the impact of HANC and OPMDs on oral health in older adults. The review entailed a descriptive‐only narrative analysis of a very narrow research field. Whilst a much larger pool of research has previously established a higher risk of oral ill‐health and deteriorated oral health‐related quality of life among HANC patients of all ages, there is a need for further research to clarify the effect of the diagnosis and management of HANC and OPMDs on oral health in older populations.

## Author Contributions

The systematic review was conceived by G.M., C.M., A.W. and F.A.; C.M. and G.M. designed the review protocol and drafted the manuscript. The primary searches were performed by C.M. with N.W. and S.A. acting as additional reviewers, contributing to screening and validating the extracted data. N.W., S.A., A.W. and F.A. reviewed the manuscript. All authors approved the final version of the manuscript.

## Funding

The authors have nothing to report.

## Ethics Statement

The authors have nothing to report.

## Consent

The authors have nothing to report.

## Conflicts of Interest

The authors declare no conflicts of interest.

## Supporting information


**Appendix S1:** Reasons for exclusion.

## Data Availability

Data sharing is not applicable to this article as no datasets were generated or analysed during this study.
